# TonB-dependent transporters and their occurrence in cyanobacteria

**DOI:** 10.1186/1741-7007-7-68

**Published:** 2009-10-12

**Authors:** Oliver Mirus, Sascha Strauss, Kerstin Nicolaisen, Arndt von Haeseler, Enrico Schleiff

**Affiliations:** 1 JWGU Frankfurt am Main, Cluster of Excellence Macromolecular Complexes, Centre of Membrane Proteomics, Department of Biosciences, Max-von-Laue Str. 9, 60438 Frankfurt, Germany; 2 Center for Integrative Bioinformatics Vienna, Max F Perutz Laboratories, University of Vienna, Medical University of Vienna, Veterinary University of Vienna, Austria

## Abstract

**Background:**

Different iron transport systems evolved in Gram-negative bacteria during evolution. Most of the transport systems depend on outer membrane localized TonB-dependent transporters (TBDTs), a periplasma-facing TonB protein and a plasma membrane localized machinery (ExbBD). So far, iron chelators (siderophores), oligosaccharides and polypeptides have been identified as substrates of TBDTs. For iron transport, three uptake systems are defined: the lactoferrin/transferrin binding proteins, the porphyrin-dependent transporters and the siderophore-dependent transporters. However, for cyanobacteria almost nothing is known about possible TonB-dependent uptake systems for iron or other substrates.

**Results:**

We have screened all publicly available eubacterial genomes for sequences representing (putative) TBDTs. Based on sequence similarity, we identified 195 clusters, where elements of one cluster may possibly recognize similar substrates. For *Anabaena *sp. PCC 7120 we identified 22 genes as putative TBDTs covering almost all known TBDT subclasses. This is a high number of TBDTs compared to other cyanobacteria. The expression of the 22 putative TBDTs individually depends on the presence of iron, copper or nitrogen.

**Conclusion:**

We exemplified on TBDTs the power of CLANS-based classification, which demonstrates its importance for future application in systems biology. In addition, the tentative substrate assignment based on characterized proteins will stimulate the research of TBDTs in different species. For cyanobacteria, the atypical dependence of TBDT gene expression on different nutrition points to a yet unknown regulatory mechanism. In addition, we were able to clarify a hypothesis of the absence of TonB in cyanobacteria by the identification of according sequences.

## Background

Filamentous cyanobacteria contain molecular machines for oxygenic photosynthesis under all growth conditions [1]. These machines, as well as those involved in respiration and nitrogen metabolism, depend on non-proteinaceous cofactors such as iron [2,3]. The level of iron found in cyanobacteria is generally one order of magnitude higher than in non-photosynthetic bacteria [4] and represents about 0.1% of their biomass [5]. Even though iron and copper are required for the function of respiratory and photosynthetic complexes, their intracellular level has to be tightly controlled as these ions pose a risk of oxidation [3]. Therefore, the uptake of iron is highly regulated in order to avoid intoxication. On the other hand, it is hypothesized that iron limitation might have been one of the selective forces in the evolution of cyanobacteria [6], and one might speculate that those cyanobacteria with the most efficient iron uptake systems might have had an evolutionary advantage. To enhance iron uptake, eubacteria secrete low-molecular-weight iron chelators (siderophores) under iron-limiting conditions to complex environmental iron [7]. The siderophore-iron complexes are bound by receptor proteins (TonB-dependent transporters, TBDTs) in the outer membrane which are composed of a transmembrane β-barrel domain, a so-called plug domain and a periplasmic exposed TonB box. The siderophore-iron is subsequently transferred to the cytoplasm by transport proteins in the cytoplasmic membrane [8,9]. This process is dependent on TonB which provides the energy required for the translocation of siderophore-iron complexes across the outer membrane [10]. In order to facilitate this translocation, the periplasmic domain of TonB interacts with the TonB box of the loaded TBDT. It is proposed that TonB exerts a pulling force on the TonB box and, thereby, partially unfolds the plug domain enabling the translocation of the siderophore into the periplasmic space [11]. Several TBDTs have been identified. Beside the ones for iron transport [12,13], TBDTs for nickel [14], disaccharides (for sucrose SuxA; [15], for maltose MalA; [16]), oligo- (CsuF; [17]), polysaccharides (SusC; [18]) or large degradation products of proteins (RagA; [19]) are described. The most intensively studied function of TBDTs is the iron uptake in Gram-negative bacteria. Three large classes are defined, namely transferrin-/lactoferrin-binding proteins, porphyrin and siderophore transporters [20]. In addition to the transport of iron across the outer membrane by TBDTs, an additional ferric iron uptake system is postulated, but the corresponding outer membrane receptor has not yet been identified [21]. The TBDTs TbpA (transferring-binding protein A) and LbpA (lactoferrin-binding protein A) facilitate the uptake of iron from transferrin/lactoferrin, respectively; the uptake is also assisted by the lipoproteins TbpB and LbpB which face the extracellular side [22]. The porphyrin-transporting TBDTs include HasR, HgbA, HmbR (heme; [12,22]) and BtuB which transports the cobalt-complexing vitamin B_12 _(cobalamin [23]). Heme uptake is especially important in bacterial pathogens, where various heme-containing compounds are utilized [13]. The siderophore TBDTs are further sub-classified according to their substrate - that is the chemical nature of the siderophore they bind. Siderophores belong inter alia to hydroxamates, catecholates, phenolates, citrates or combinations thereof [9]. For example, the siderophore transporters FepA, ViuA and IroN recognize catecholates, FhuA, FoxA and FhuE hydroxamate and FecA citrate.

The iron uptake system in cyanobacteria is not well understood. For the non-filamentous cyanobacterium *Synechocystis *sp. PCC 6803 the TBDTs encoded by *sll1206, sll1406, sll1409 *and *slr1490 *were partially characterized [24,25]. For filamentous cyanobacteria such as *Anabaena *sp. PCC 7120 (also termed *Nostoc *sp. PCC 7120) only siderophore secretion [26-28], and the influence of enhanced or reduced iron levels on the growth [29-32], were investigated. *Anabaena *sp. PCC 7120 secretes the hydroxamate-type siderophore schizokinen, allegedly the only siderophore secreted [26,27]. Only recently, a TBDT encoded by *schT (alr0397*) involved in the uptake of schizokinen was identified. The expression of the gene *schT *(*alr0397*) was mildly increased under a shortage of Fe^3+^. A *schT *knock-out mutant showed a moderate phenotype of iron starvation, and the characterization of its siderophore-dependent iron uptake demonstrated the function of *schT *as a TonB-dependent schizokinen transporter [33].

To learn more about iron transport systems in general and in cyanobacteria particularly we searched for genes coding for TBDTs based on previously experimentally characterized TBDTs. Subsequently, we assigned putative substrates for so far uncharacterized TBDTs, according to their sequence similarity to already known TBDTs. We observed a substantial difference in the number of TBDT genes in the analysed cyanobacteria. The expression pattern of the TBDT genes in *Anabaena *sp. PCC 7120 is analysed with respect to iron, copper and nitrogen availability.

## Results and discussion

### Classification of TonB-dependent transporters

Ninety-eight TBDTs and the (putative) substrates (for example, metallophores or sugars) were extracted from the published literature (see Additional file 1) [14-19,22,34-124]. In order to classify the TBDTs with unknown substrates, we first searched for putative TBDTs in 686 sequenced genomes. We identified 4600 putative TBDTs in 347 species (see Additional file 2). Compared to previously published bioinformatic analyses [15,125], we identified fewer sequences in the species which had been analysed in the past due to a more stringent cutoff (not shown). More specifically, within the species analysed by Koebnik, we selected seven sequences not previously identified, but did not consider 103 sequences [125]. A similar ratio was found when analysing the number of sequences selected by us from the species analysed by Blanvillain *et al*. (+22/-142; [15]), who selected 3020 sequences which resulted in a discrepancy of about 5%.

**Figure 1:**
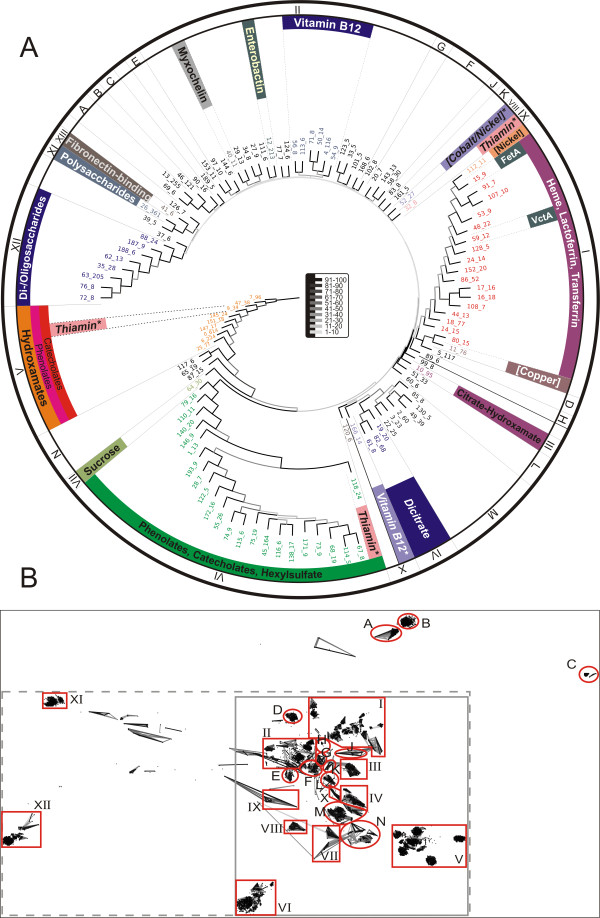
**Clustering of the sequences of putative TonB-dependent transporters (TBDTs)**. The sequences found by the described genome-wide searches were analysed by CLANs as depicted. (A) shows the consensus tree of the pair-wise mean cluster distances. The branches are coloured according to their respective bootstrap value in shades of grey as indicated by the legend in the middle of the tree. The numbers at each leaf are of the format 'x_y', where 'x' is the cluster number and 'y' the number of sequences belonging to this cluster. We have further indicated the transported substrates and the regions as shown in Figure 1B are marked by I to XII and A to N. Brackets indicate that the metal ion is known, but the metallophore has not yet been identified. An asterisk marks predicted substrates. (B) shows the result of two-dimensional clustering in CLANS. The regions from Figure 1A are marked by red polygons (containing at least a single exp/pTBDT) and red circles (no functionally characterized TBDT). Sequences with a high similarity (*P*-value < 10^-90^) are connected by lines coloured in shades of grey (the darker the smaller the *P*-value). The regions shown in Figure 2 (grey dashed line) and Figure 3 (grey dashed-dotted line) are highlighted.

**Figure 2:**
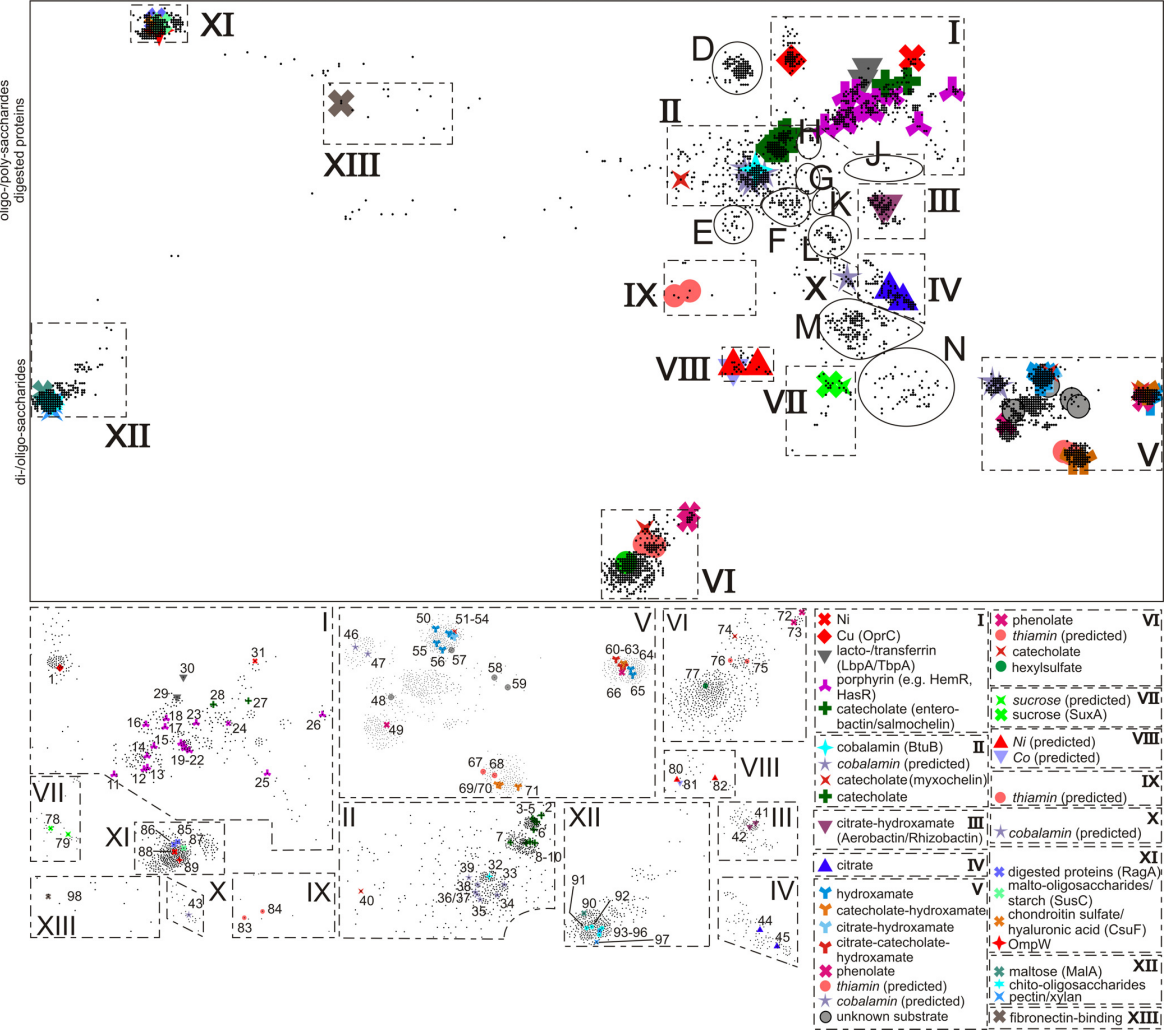
**Distribution of the sequences of characterized (experimental/predicted) TonB-dependent transporters (TBDTs)**. The grey dashed frame from Figure 1B is shown here containing expTBDTs and pTBDTs, which are marked by colored symbols and a number, which corresponds to the numbering in column 1 (see Additional file 1, [14-19,22,34-124]). Symbols are used to enhance the readability of the figures and are explained on the bottom right. The dashed frames are shown in a magnified view on the bottom. Circles define regions without functionally characterized TBDTs. For regions XI and XII the substrates are indicated on the left. The region numbering is explained in the text.

We subsequently performed a cluster analysis of the identified sequences of putative TBDTs (see Methods) leading to 195 clusters with at least two sequences. Figure 1A shows the consensus tree used to highlight 'regions' on the two-dimensional sequence landscape. A region is marked by roman numerals if the substrate for at least one TBDT in this region is experimentally verified (expTBDTs) or predicted (pTBDTs), and marked by upper case letters if no substrate TBDT in the region is known (Figure 1). Figure 1B shows the expTBDT regions I-VII, XI, XII and XIII and the pTBDTs regions VIII, IX and X together with the uncharacterized regions A-N. Figure 2 shows an enlarged version of the dashed rectangle in Figure 1B. The colours describe the substrate that binds to the corresponding TBDTs. Figure 2 (bottom) shows a magnification of the expTBDTs regions, where the numbers refer to sequences with a known substrate (Additional file 1, [14-19,22,34-124]). In the following, we characterize the regions according to the 98 TBDTs that have been experimentally verified or predicted (Additional file 1, [14-19,22,34-124]).

### Region I

This consists of 19 clusters with at least five sequences and 10 of them have assigned functions which include porphyrin, lacto-/transferrin and nickel transporters (Figure 2). Cluster 11 contains the sequence of the copper chelate binding protein OprC (sequence 1, for references see Additional file 1, [14-19,22,34-124]). A group of clusters (15, 17, 18, 59, 86 and 107; sequences 11-28) are comprised of heme-transporting (HmbR) proteins. Remarkably, the two enterobactin (catecholate; see Additional file 1, [14-19,22,34-124]) transporters VctA (cluster 59; sequence 28) and FetA (cluster 15; sequence 27) are located within the porphyrin group. This finding corroborates the observation that VctA and FetA are, supposedly, involved in transporting porphyrin [126,127]. In cluster 16, the LbpA or TbpA proteins are found (sequences 29, 30). There is also a small cluster (112) which contains nickel-transporting TBDTs with a single expTBDT (sequence 31, FrpB4).

### Region II

This contains 20 clusters, three of them represented by expTBDTs (cluster 4, 12, 40). In cluster 4 the experimentally confirmed cobalt-complexing vitamin B_12 _transporter BtuB (sequence 32) is present. However, in the same cluster, and in clusters 160 and 165, predicted BtuBs were identified (sequences 33-39). Cluster 12 contains IrgA, BfrA or IroN sequences (No. 2-10) transporting enterobactin, DHBS (catecholate) or salmochelin (glycosylated catecholate). In addition, a myxochelin (catecholate) transporter (sequence 40, cluster 40) occurs in region II.

### Region III

Cluster 10 is defined by sequences of the aerobactin/rhizobactin (Citrate-hydroxamate; see Additional file 1, [14-19,22,34-124]) transporters IutA and RhtA (sequence 41 and 42).

### Region IV

The largest cluster in region IV (No. 82) contains the sequences of the ferric rhizoferrin (carboxylate) transporter RumA and the diferric dicitrate transporter FecA (sequences 44 and 45).

### Region V (sequences 46-71)

This consists of nine clusters with three (clusters 0, 6, 7) of them containing expTBDTs and two pTBDTs (clusters 9, 25). This region mainly contains transporters for hydroxamate-type siderophores, such as desferrioxamine (hydroxamate; cluster 0, sequences 50, 51, 56), ferrichrome (hydroxamate; cluster 0, sequence 55), pseudobactin A (citrate-catecholate-hydroxamate; cluster 6, sequences 62, 63), pyochelin (phenolate; cluster 6, sequence 66), or anguibactin (catecholate-hydroxamate; cluster 7, sequences 69-71). Interestingly, the proteins for which sequences are found in cluster 9 (sequence 67, 68) are predicted to transport thiamin, whereas proteins 46 and 47 (cluster 25) are predicted to transport vitamin B_12_. The latter appears to be a false prediction as judged from the large distance to cluster 4 containing the experimentally confirmed BtuB. Setting an even lower *P*-value (1E-100 instead of 1E-90) as the threshold for defining the clusters in CLANS leads to cluster 0 splitting up in the upper part with all hydroxamate-type TBDTs (including all cyanobacterial TBDTs of cluster 0) and the lower part containing phenolate-transporting TBDTs and VciA, which has been shown to transport neither heme, vibriobactin, enterobactin, ferrioxamine B, aerobactin nor shizokinen [77].

### Region VI

This region represents transporters for phenolates, catecholates or hexylsulfate and contains several clusters. A hexylsulfate transporting TBDT (sequence 77) can be found in cluster 45, a vibriobactin (catecholate) transporter (sequence 74) in cluster 140 and proteins transporting yersiniabactin (phenolate; sequences 72, 73) in cluster 79. As already observed in region V, we also detected two sequences (75, 76) in cluster 118 that are putative thiamin transporters.

### Region VII (cluster 67)

Cluster 67 contains SuxA (sequence 78), an experimentally verified sucrose transporter. Please note, that sequence 79 has been predicted to transport sucrose [15]. The prediction was based on the co-localization of the corresponding gene with the transcriptional regulator ScrR. Thus, our bioinformatic analysis provides additional evidence for the functional characterization.

### Region VIII (cluster 52)

This region contains predicted nickel and cobalt TBDTs with unknown metallophore specificity and no representative of the expTBDTs.

### Region IX

This consists of eight sequences in one cluster (No. 32), where two of the eight are putative thiamin transporters. However, proteins assigned as thiamin transporters were also found in regions V (sequences 67, 68, cluster 7) and VI (sequences 75 and 76, cluster 118). Their genes are co-localized on the genome with a cytoplasmic membrane transporter for thiamin (PnuT, [128]), however, the functional assignment remains to be proven.

### Region X

This contains a TBDT predicted to transport cobalt-complexing vitamin B_12 _(sequence 43, cluster 166). However, it is far away from the BtuB cluster in region II (Figure 2). Hence, the assigned function should be experimentally confirmed.

### Region XI

The region is clearly separated from the rest and contains cluster 26. The experimentally characterized TBDTs include oligosaccharide (CsuF, sequence 88), polysaccharide transporters (SusC, sequence 87) and transporters for degradation products of proteins (RagA, sequences 85-86). While many taxa are represented by sequences in the region I-X, region XI consists almost exclusively of bacteroidetes with the exception of one δ-proteobacterial sequence (gi|108757959, *Myxococcus xanthus*). Thus, sequences in this region may indicate a special adaptation of these organisms, which may be due to their lifestyle. Bacteroidetes are involved in food digestion in the intestinal tract of mammals. Hence a specific TBDT class for the uptake of substrates provided by the host seems plausible.

### Region XII

This also appears as an outlier (Figure 2). It contains eight clusters (35, 62, 63, 72, 76, 88, 187 and 188) and only one expTBDT MalA (sequence 90, cluster 63) that transports maltodextrin. Seven pTBDTs are said to transport xylan, pectin or chito-oligosaccharides (No. 91-97; for references see Additional file 1, [14-19,22,34-124]), where sequences 91-96 belong to cluster 63 and sequence 97 to cluster 72. It appears that this region is composed of di- and oligosaccharide transporters. In line with this notion, the δ-proteobacterial TBDTs are from Myxobacteria (*Myxococcus xanthus*, *Sorangium cellulosum*), which are found on decaying plant material consuming their saccharides. Most of the sequences in this region stem from α- and γ-proteobacteria (18.4%, 76%) and a few bacteroidetes, δ- and β-proteobacteria taxa.

### Region XIII

Positioned between region XI and the crowded area on the right side, this region is defined by a fibronectin-binding TBDT (sequence 98, cluster 41). As in most of the sequences in region XI, the sequences of this region consist almost exclusively of bacteroidetes. The close proximity of regions XI and XIII is consistent with the observed interaction of the TBDT with a glycoprotein.

### Other regions

For regions I to XIII we were in the lucky position of being able to infer at least putative functions to ~3700 sequences. (The putative annotation can be viewed at .) However, from the 4600 sequences from GenBank ~900 sequences remain in regions A-N, where we were unable to assign any function (Figure 1). While we cannot discuss potential substrates for clusters in regions A-N, we can at least point to some regions that show a peculiar taxonomic composition. In regions A and B sequences from mostly γ- (74%) and α-proteobacteria (19%), but also a few β-proteobacterial (5%) and bacteroidetes (1.5%), are present. Region C contains exclusively γ-proteobacterial sequences.

### Classification of TonB-dependent transporters in cyanobacteria

**Figure 3:**
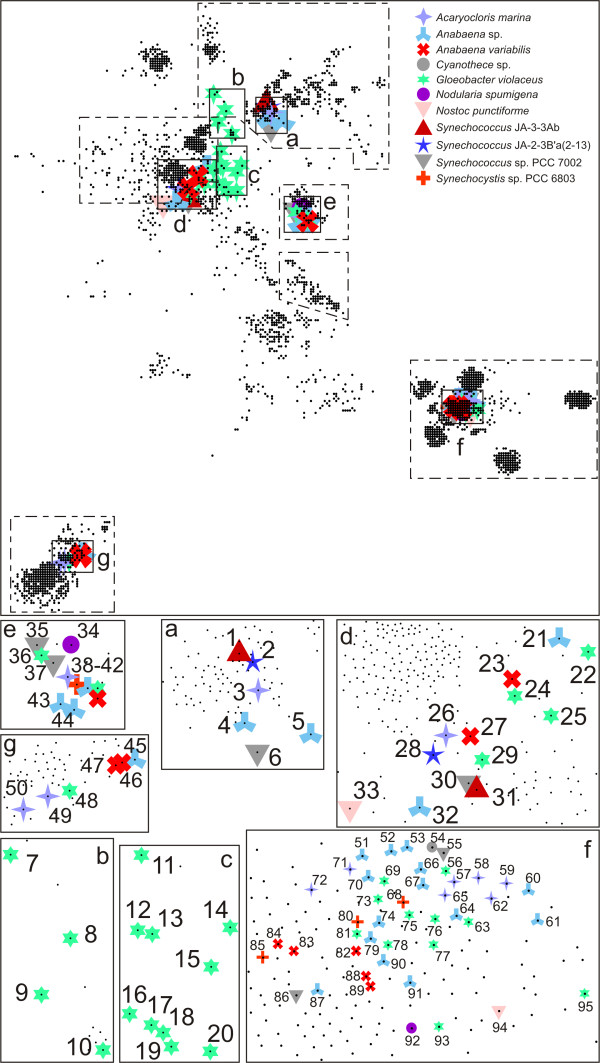
**Distribution of TonB-dependent transporters (TBDTs) found in genomes of cyanobacteria**. Cyanobacterial sequences of TBDTs are highlighted and the containing frames are enlarged at the bottom. Dashed boxes indicate the dimensions shown in Figure 1. The colour code shows the different species as indicated in the right corner. The numbers are according to Table 1.

**Figure 4:**
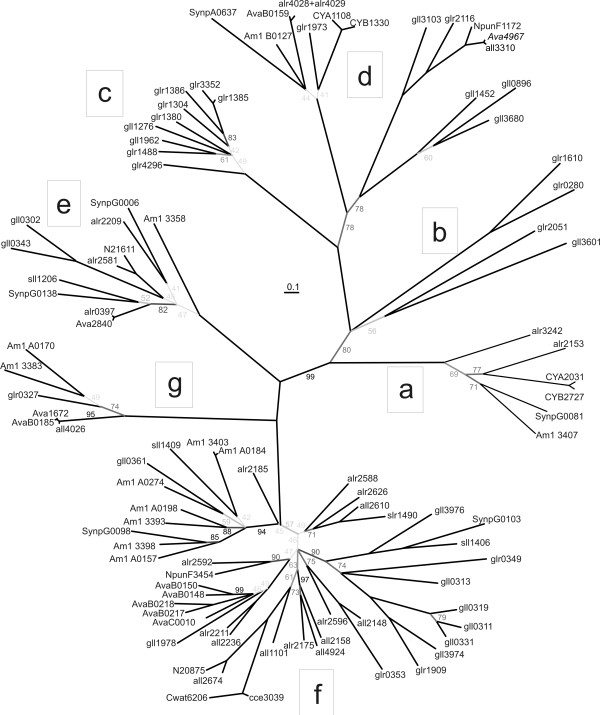
**Distribution of cyanobacterial sequences**. An alignment of sequences of TonB-dependent transporters listed in Table 1 was used to reconstruct a maximum likelihood phylogeny. Bootstrap values were calculated from 1000 phylogenetic trees. To indicate the probability of occurrence of an edge in these trees the edges are shown in shades of grey.

One of our aims was the identification and classification of cyanobacterial TBDTs. Hence we searched for sequences of putative TBDTs in 32 cyanobacterial genomes (proteins listed according to their accession code (Table 1, column 1). We additionally extracted the automated annotation from GenBank (Table 1, column 2). At present, this annotation is mostly limited to CirA, FhuE or BtuB. Hence, we analysed the location of cyanobacterial sequences on the CLANS plot (Figure 3 shows the section of Figure 1B indicated by a grey box). All cyanobacterial TBDTs belong to regions with experimentally characterized TBDTs (see Figure 2 and dashed frames in Figure 3). To further confirm the classification determined with CLANS we also constructed a phylogenetic tree for the cyanobacterial sequences (Figure 4). Seven 'subtrees' (a-f) were identified and mapped to regions I-X.

**Table 1:** Sequences used for the phylogenetic analysis of cyanobacteria

**Code**	**Old**	**New**	**Spot**	**Database code**	**Species**
Am1 B0127	BtuB	BtuB	26	gi|158339996	*Acaryochloris marina MBIC11017*
	
Am1 3407	CirA	HutA	3	gi|158336543	
	
Am1 3358	CirA	IutA	38	gi|158336494	
	
Am1 A0170	CirA	ViuA	50	gi|158339820	
	
Am1 3383	CirA	ViuA	49	gi|158336519	
	
Am1 A0184	Fiu	FhuA	58	gi|158339834	
	
Am1 A0274	FhuE	FhuA	57	gi|158339535	
	
Am1 A0198	-	FhuA	71	gi|158339845	
	
Am1 A0157	FhuE	FhuA	59	gi|158339807	
	
Am1 3393	FhuE	FhuA	72	gi|158336529	
	
Am1 3398	-	FhuA	65	gi|158336534	
	
Am1 3403	Fiu	FhuA	62	gi|158336539	

Ava_B0148	FhuE	FhuA	89	gi|75812430	*Anabaena variabilis *ATCC 29413
	
Ava_B0150	CirA	FhuA	88	gi|75812432	
	
Ava_B0159	BtuB	FhuA	82	gi|75812441	
	
Ava_B0185	CirA	ViuA	46	gi|75812467	
	
Ava_B0217	Fiu	BtuB	23	gi|75812499	
	
Ava_B0218	CirA	IutA	42	gi|75812500	
	
Ava_C0010	FhuE	BtuB	27	gi|75812671	
	
Ava_1672	CirA	ViuA	47	gi|75907894	
	
Ava_2840	CirA	FhuA	83	gi|75909052	
	
Ava_4967	BtuB	FhuA	84	gi|75911163	

cce3039	CirA	FhuA	54	gi|172037952	*Cyanothece *sp. CCY0110

glr0280	CirA	?	7	gi|37519849	*Gloeobacter violaceus *PCC7421
	
gll0302	CirA	IutA	41	gi|37519871	
	
gll0311	-	FhuA	81	gi|37519880	
	
gll0313	-	FhuA	56	gi|37519882	
	
gll0319	-	FhuA	63	gi|37519888	
	
glr0327	CirA	ViuA	48	gi|37519896	
	
gll0331	FecA	FhuA	69	gi|37519900	
	
gll0343	CirA	IutA	36	gi|37519912	
	
glr0349	FhuE	FhuA	95	gi|37519918	
	
glr0353	-	FhuA	76	gi|37519922	
	
gll0361	FhuE	FhuA	78	gi|37519930	
	
gll0896	FepA	BtuB	22	gi|37520465	
	
gll1276	OMC	BtuB	20	gi|37520845	
	
glr1304	OMC	BtuB	17	gi|37520873	
	
glr1380	-	-	-	gi|37520949	
	
glr1385	-	BtuB	18	gi|37520954	
	
glr1386	CirA	BtuB	15	gi|37520955	
	
gll1452	BtuB	BtuB	25	gi|37521021	
	
glr1488	-	BtuB	12	gi|37521057	
	
glr1610	-	?	8	gi|37521179	
	
glr1909	FhuE	FhuA	73	gi|37521478	
	
gll1962	-	BtuB	16	gi|37521531	
	
glr1973	BtuB	BtuB	29	gi|37521542	
	
gll1978	Fiu	FhuA	93	gi|37521547	
	
glr2051	FepA	?	10	gi|37521620	
	
glr2116	BtuB	BtuB	24	gi|37521685	
	
gll3103	BtuB	BtuB	11	gi|37522672	
	
glr3352	OMC^a^	BtuB	19	gi|37522921	
	
gll3601	BtuB	BtuB	14	gi|37523170	
	
gll3680	BtuB	?	9	gi|37523249	
	
gll3974	FhuE	FhuA	77	gi|37523543	
	
gll3976	CirA	FhuA	75	gi|37523545	
	
glr4296	CirA	BtuB	13	gi|37523865	

NpunF1172	-	BtuB	33	gi|186681644	*Nostoc punctiforme *PCC73102
	
NpunF3454	-	FhuA	94	gi|186683610	

all1101	-	FhuA	52	gi|17228596	*Anabaena *sp. PCC 7120
	
all2148	FhuE	FhuA	66	gi|17229640	
	
all2158	FhuE	FhuA	53	gi|17229650	
	
all2236	Fiu	FhuA	87	gi|17229728	
	
all2610	CirA	FhuA	67	gi|17230102	
	
all2674	Fiu	FhuA	70	gi|17230166	
	
all3310	BtuB	BtuB	21	gi|17230802	
	
all4026	CirA	ViuA	45	gi|17231518	
	
all4924	FhuE	FhuA	61	gi|17232416	
	
alr0397	CirA	IutA	40	gi|17227893	
	
alr2153	CirA	HutA	4	gi|17229645	
	
alr2175	-	FhuA	69	gi|17229667	
	
alr2185	Fiu	FhuA	51	gi|17229677	
	
alr2209	CirA	IutA	44	gi|17229701	
	
alr2211	CirA	FhuA	90	gi|17229703	
	
alr2581	CirA	IutA	43	gi|17230073	
	
alr2588	CirA	FhuA	74	gi|17230080	
	
alr2592	FhuE	FhuA	91	gi|17230084	
	
alr2596	FhuE	FhuA	64	gi|17230088	
	
alr2626	-	FhuA	79	gi|17230118	
	
alr3242	CirA	HutA	5	gi|17230734	
	
alr4028 +alr4029	-	BtuB	32	gi|17231520 gi|17231521	

sll1206	IutA	IutA	39	gi|16329186	*Synechocystis *sp. PCC6803
	
sll1409	FhuA	FhuA	80	gi|16329191	
	
sll1406	FhuA	FhuA	85	gi|16329194	
	
slr1490	FhuA	FhuA	68	gi|16329201	

CYA_1108	BtuB	BtuB	31	gi|86605797	*Synechococcus sp*. JA-3-3Ab
	
CYA_2031	CirA	HutA	1	gi|86606671	

CYB_1330	BtuB	BtuB	28	gi|86608804	*Synechococcus sp*. JA-2-3B'a(2-13)
	
CYB_2727	CirA	HutA	2	gi|86610153	

SynpA0637	BtuB	BtuB	30	gi|170077260	*Synechococcus sp. PCC 7002*
	
SynpG0081	CirA	HutA	6	gi|170076551	
	
SynpG0006	CirA	IutA	35	gi|170076476	
	
SynpG0138	CirA	IutA	37	gi|170076608	
	
SynPG0089	FhuE	FhuA	55	gi|170076568	
	
SynpG0103	FhuA	FhuA	86	gi|170076573	

Nspu20875	CirA	FhuA	92	gi|119508873	*Nodularia spumigena* CCY9414
	
Nspu21611	CirA	IutA	43	gi|119509643	

Cwat6206*	-	FhuA	1	gi|67920343	*Crocosphaera watsonii *WH8501

The annotation of the sequences is indicated in column 1, the spot number according to Figure 3 is indicated in column 4; the initial annotation in the database is given in column 2; the classification according to Figure 3 using the name of a representative transporter of the related category is given in column 3; the accession code in column 5; and the source organism column 6.

^a^(OMC, outer membrane channel; '*', incomplete sequence; '?', no clear assignment possible).

The six sequences in subtree 'a' belong to region I (Figure 3, 4) and show a relation to heme transporters such as HutA (Figures 1, 2, sequence 13). The sequences are found in *Synechococcus *sp.*, Acaryochloris marina *and *Anabaena *sp. PCC 7120 (see new assignment in Table 1, column 3). Subtrees 'b' and 'c' contain only sequences from *Gloeobacter violaceus*. Subtree 'b' is within region I and is equidistant to enterobactin and heme transporters. Thereby, a clear assignment to a characterized TBDT family appears currently impossible. Subtree 'd' is close to the BtuB transporter cluster (region II) (Figure 1). In this region we find sequences from most of the analysed cyanobacteria (8 of 12), suggesting that transporters with similarity to BtuB are common. Subtree 'e' (Figure 3, 4) represents transporters, which can clearly be assigned as specific for aerobactin/rhizobactin (IutA-/RhtA-type). Subtree 'f' represents sequences of transporters with the closest relation to FhuA-type transporters of cluster 0. The sequences of subtree 'g' (cluster 1), closely related to ViuA, are probably transporters for catecholates. The sequences of subtree 'g' are also close to cluster 118, which contains putative thiamin transporters. Nevertheless, since the two putative thiamin transporters have not yet been experimentally confirmed, we consider these cyanobacterial TBDTs to be iron transporters of the ViuA-type.

Summarizing, the assignment of the cyanobacterial TBDTs to regions with functional characterization was successful with the exception of some TBDTs from *Gloeobacter violaceus *(subtrees 'b' and 'c'). Although BtuB-like transporters and hydroxamate-type metallophore transporters were found in cyanobacteria, we did not find FecA-type (diferric dicitrate) TBDTs, even though they occur in α-, β-, γ-, δ- and ε-proteobacteria, bacteroidetes and spirochaetes.

### Identification of TBDTs in *Anabaena *sp. PCC 7120

**Figure 5:**
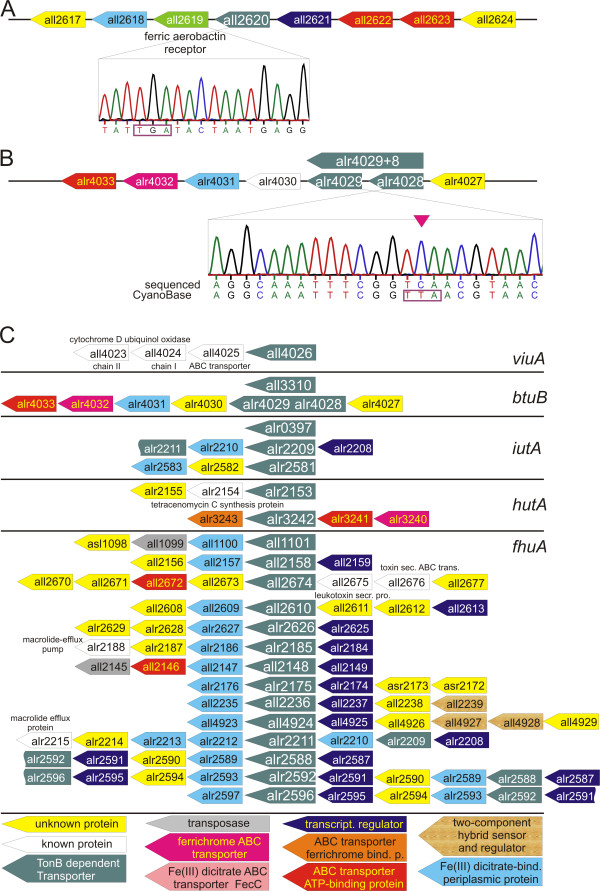
**The genomic structure of the loci coding for TonB-dependent transporters (TBDTs) in *Anabaena *sp. PCC 7120**. For A-C, the genomic structure was excised from Cyanobase ([148]) and the nomenclature of the colour code is given in C. White boxes indicate genes for which the name is given in the figure. (A) Shows the genomic orientation surrounding *all2620 *(top) and the sequencing profile of the region coding for the stop codon (in reverse orientation, bottom). (B) Shows the genomic orientation surrounding *alr4028 *(top) and the sequencing profile of the region coding for the stop codon (in reverse orientation, bottom). (C) The genomic organization surrounding the 22 other genes coding for TBDTs.

In order to explore the cyanobacterial TBDTs in more detail we analysed the full genome of *Anabaena *sp. PCC 7120. We identified 21 TBDT genes carrying the plug domain and β-barrel domain characteristic for TBDTs. In addition, we identified four genes (*all2620, alr2179, all2578, alr4028*) containing the plug domain of the TBDT, but an incomplete β-barrel domain. Downstream of *all2620 *(Figure 5A) and *alr4028 *(Figure 5B) a gene coding for the 'missing part' of the β-barrel domain is present (*all2619 *and *alr4029*, respectively). Consequently, we checked the stop codon separating the two gene pairs. We confirmed the stop codon between *all2620 *and *all2619 *(Figure 5A) and could not identify a frame shift in the sequence of the region 500 bp upstream or downstream of the stop codon. If All2620 is, indeed, part of a TBDT it has to form a heterodimer. A putative interaction partner would be All2619. It would, therefore, be interesting to investigate the existence of such complex and to understand whether it is just a remnant of a genetic accident which led to a split of the TBDT gene in *all2620 *and *all2619*. In contrast to *all2620 *and *all2619*, for *alr4028 *and *alr4029 *we found a T to C exchange in the sequence when comparing our results with that of the deposited sequence. Hence, we conclude that the stop codon does not exist and that the two genes *alr4028 *and *alr4029 *encode one protein. Therefore, 22 TBDTs exist in *Anabaena *sp. PCC 7120.

For 19 TBDTs the genomic organization suggests the integration of the gene in an operon (Figure 5C). Twelve TBDTs are directly positioned behind a gene coding for a (putative) transcriptional regulator (Figure 5C, violet), and most of the (putative) operon structures contain genes coding for proteins involved in iron transport. The gene coding for a ViuA-type transporter is in a putative operon with subunits of a cytochrome D ubiquinol oxidase, which is rather unexpected, because, to date, a relation between this oxidase and iron transport has not been reported (Figure 5C). Of the BtuB transporters one is a single gene (*all3310*), whereas the other (the gene which we confirmed and which is still annotated as *alr4028/alr4029*) is in a rather typical genomic environment, namely in front of three genes encoding the periplasmic and the plasma membrane localized iron transport machinery. The same holds true for the *hutA*-like gene *alr3242*. The other *hutA*-like gene (*alr2153*) is in a putative operon with a gene encoding a tetracenomycin C synthesis protein and a gene of unknown function. Again, the relation between the TBDT and the downstream genes are rather questionable.

Three genes are classified as *iutA*-like. *alr0397 *(*schT*) is single standing in the genome. Downstream of *alr2581 *we found two genes coding for an unknown protein and a dicitrate binding protein, respectively. *Alr2209 *is a component of a large genomic region (~14 kbp, *alr2208-alr2215*) containing upstream a transcription regulator and downstream a cluster with three genes coding for periplasmic dicitrate binding proteins and one *fhuA*-like gene (*alr2211*). Thirteen of the 14 *fhuA*-like genes are upstream of a gene coding for a protein annotated as dicitrate-binding. However, most of the genes found in the putative operons defined by the 14 *fhuA*-like genes encode for proteins of unknown function. Three of the *fhuA*-like genes (*alr2588, alr2592, alr2596*) are in the same chromosomal region. Upstream of these, a gene coding a transcription regulator and downstream a gene encoding a dicitrate binding protein are found. However, the phylogenetic analysis (Figure 4) argues against recent gene duplication.

### Variations of the number of genes encoding TBDTs in cyanobacteria

The results presented in Figures 3, 4, 5 and Table 1 show that the number of TBDTs varies among cyanobacteria. We found 22 TBDTs in *Anabaena *sp. PCC 7120, 10 in *Anabaena variabilis*, six in *Synechococcus *sp. PCC 7002, four in *Synechocystis *sp. PCC 6803, 33 in *Gloeobacter violaceus*, but no TBDTs in the genomes of, for example, *Prochlorococcus*). This variation of the number of genes, however, does not reflect an elevated amount of outer membrane protein coding genes in *Anabaena *sp. PCC 7120, because such a variation is not found for other outer membrane proteins (Omp85, TolC, OstA and others; not shown). Furthermore, in a previous report, a correlation of the number of TBDTs to the number of open reading frames as, for example, for transporters in the cytoplasmic membrane [129] was not observed [128], which is supported by our analysis (not shown).

TBDTs are regulated by TonB proteins. Hence, the large number of TBDTs leads to the question of whether each TBDT is regulated individually or (at least a sub-population of the TBDTs) in concert by one TonB protein. We, therefore, screened the genomes for the presence of *tonB *(Table 2). One to three *tonB *genes were detected. Hence, the number of TBDTs largely exceeds the number of TonB proteins. Please note that we identified a TonB-like protein (Slr1484) in *Synechocystis *sp. PCC 6803, which corrects a previous statement excluding the presence of a TonB-like protein in this species [130].

**Table 2:** TonB-like genes in cyanobacteria

**species**	**No. TonBs**	**Locus tag**
*Acaryochloris marina *MBIC11017	2	AM1_A0167, AM1_3413

*Anabaena variabilis *ATCC 29413	1	Ava_2295

*Crocosphaera watsonii *WH 8501	1	CwatDRAFT_6356

*Cyanothece *sp. CCY0110	2	CY0110_08196, CY0110_24616

*Gloeobacter violaceus *PCC 7421	3	glr1389, glr1815, glr2404

*Nodularia spumigena *CCY9414	1	N9414_10453

*Nostoc punctiforme *PCC 73102	1	Npun_F0783

*Anabaena *sp. PCC 7120	1	all5036

*Synechococcus *sp. PCC 7002	2	SYNPCC7002_G0090, SYNPCC7002_A2465

*Synechococcus *sp. JA-3-3Ab	1	CYA_2030

*Synechococcus *sp. JA-2-3B'a(2-13)	1	CYB_2726

*Synechocystis *sp. PCC 6803	1	slr1484

### Expression of genes in *Anabaena *sp. coding for TBDTs

**Figure 6:**
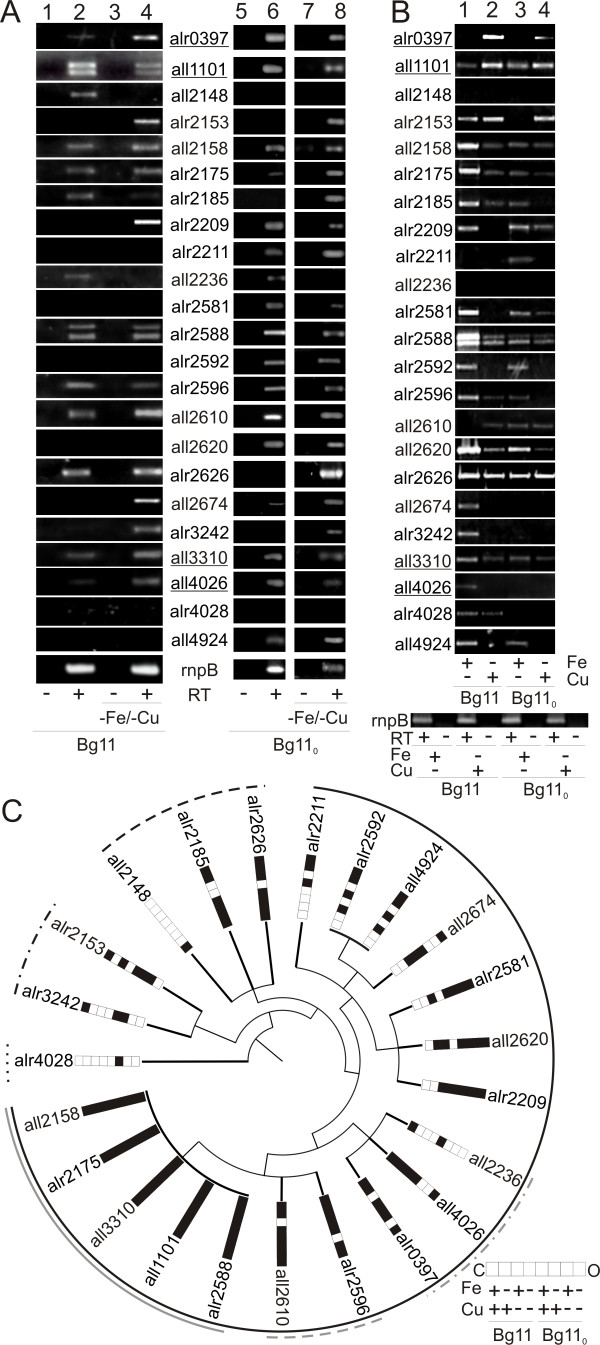
**Iron-dependent expression of TonB-dependent transporters (TBDTs) of *Anabaena *sp. PCC 7120**. (A)* Anabaena *sp. PCC 7120 were grown in media with (lane 1, 2, 5 and 6) and without a source of ferric ammonium citrate and copper (-Fe/-Cu, lane 3, 4, 7 and 8) as well as with (BG11, lane 1-4) and without a source of nitrogen (BG11_0_, lane 5-8). RNA was isolated and RT-PCR was performed to visualize expression with (+) and without (-) addition of reverse transcriptase using primers listed in Table 3. The transcript of *rnpB *was amplified as control. (B)*Anabaena *sp. PCC 7120 were grown in media with (BG11, lane 1, 2) or without nitrogen source (BG11_0_, lane 3, 4) in the absence of either copper sulfate (lane 1, 3) or ferric ammonium citrate (lane 2, 4) and RNA isolated from 50 ml cells of log phase cultures as described. RT-PCR was performed to visualize expression with and without addition of reverse transcriptase using primers listed in Table 3. (C) The results from A and B are visualized as bar code where black stands for detected expression, and white for no expression detected under conditions used. The order is given on the right (C stands for: pointing to the centre). The black outer circle marks all genes expressed in BG11_0_, the black dashed line all genes expressed in BG11, the black dashed dotted line all genes not expressed without starvation and the dotted line the gene only weakly expressed upon copper limitation in BG11. The grey line on the outer rim indicates all genes always expressed, the grey dashed line on the outer rim all genes where expression was not detected under one condition and the grey dashed dotted line on the outer rim all genes which are expressed in BG11 and BG11_0_, but not in all other media.

We analysed the gene expression of the 22 TDBT genes and of *all2620*, which only codes for the N-terminal portion of a TBDT (Figure 5A) in *Anabaena *sp. PCC 7120 (Figure 6). To this end, *Anabaena *sp. PCC 7120 was grown in normal medium (BG11), medium without iron (BG11_-Fe_), medium without copper (BG11_-Cu_) or medium lacking both (BG11_-Fe-Cu_). The presence of transcript was then determined by non-quantitative reverse transcription polymerase chain reaction (RT-PCR; primers are listed in Table 3). Iron and copper were chosen, because iron is known to be involved in the regulation of the gene expression of TBDTs and copper was recently found to induce an expression of a gene cluster involved in siderophore synthesis [131]. Remarkably, 13 TBDT-gene transcripts were present under normal growth conditions in such amounts that they could be amplified and visualized by RT-PCR (Figure 6A, lane 2; Figure 6C, grey lines and black dashed line). It should be noted that the absence of a transcript for the other genes might only reflect low transcript abundance. For 19 genes, we detected transcripts under Fe minus or/Cu minus conditions (Figure 6A lane 4, 6B, lane 1, 2). The analysis of the detection pattern revealed the following: (1) the genes *all2148 *and *all2236*, both hydroxamate-type TBDTs, were down-regulated upon iron and/or copper starvation compared to transcript levels under normal conditions; (2) the expression of seven genes (*iutA*-like genes *alr2209 *and *alr2581*, the *btuB*-like gene *alr4028*, the *hutA*-like gene *alr3242 *and the *fhuA*-like genes *all2674, all4924 *and *alr2592*) not detected under normal growth conditions is increased in response to copper, but not iron, limitation in the BG11 medium (Figure 6B, lane 1). This is notable, because, for four of these seven genes, the expression in the absence of one metal-ion (either Cu or Fe) is higher than in the absence of both iron and copper. One *viuA*-like gene (*all4026*) is expressed at a low level in BG11, but not in BG11 deficient of iron. An exclusive dependence of (upregulation of) expression in BG11 medium on iron limitation was only observed for *alr0397 *(*iutA*-like) and *all2610 *(*fhuA*-like).

**Table 3:** Primers used for reverse transcription polymerase chain reaction analysis and sequence confirmation.

**Use**	**Primer**	**Oligonucleotide sequence**
RT-PCR	all1101-F	CGCTGCTCTATCGCCTGACTG
	
	all1101-R	GCGTACTTTCCAGCGATTGTGC
	
	all2148-F	ACGGTGACGGGGGAAACAGGA
	
	all2148-R	ACTTCCACTCGCTCAATCGTCC
	
	all2158-F	CACCACCAGCAGAACCAACAGC
	
	all2158-R	CGATAAATCCACCTCACCACGG
	
	all2236-F	AATCGCGCGGCACTCTACCGT
	
	all2236-R	GGATAACTCCAATCCCACGAGC
	
	all2610-F	CAGTCATAGGAGAGGCGGGATT
	
	all2610-R	CATAGAGTACAGAAGCCGGTCC
	
	all2620-F	AAACTCCCTCAACCGCGCTGG
	
	all2620-R	GGAATCCGCGAACCATCGGC
	
	all2674-F	CCCAGAAACACCTACCGCAGAA
	
	all2674-R	AACTAGGTTGACGACCCCACCT
	
	all3310-F	TTTAGGCAACCCAGGCGGCAC
	
	all3310-R	GCATTATGCTCAAAGGTGACGCG
	
	all4026-F	GTGGTTTTTGTGGAGTGTGGGG
	
	all4026-R	CCATCAACTGGTGTGTCTTCCC
	
	all4924-F	CCCTACCAGAGGATCTGGGGA
	
	all4924-R	CTGTACCAACGGCTGGTAAGAC
	
	alr0397-F	TGCGTCGCGGGATTTGCGAAC
	
	alr0397-R	GGATAGTATTGACCCTGGGGTC
	
	alr2153-F	CCTCCCGTGGGATTAACTTTGG
	
	alr2153-R	CGCATCAGGGCCCACTCGA
	
	alr2175-F	GGTGTCCCGGCTGTTGGTACT
	
	alr2175-R	AGTACCTCAAACCTCTCTGGGC
	
	alr2185-F	CCCCGTCAGGTACTCGAAGAC
	
	alr2185-R	TCTTCCATGTCAAGCTAGGGGC
	
	alr2209-F	CCTACTCCCACACCCCCAAC
	
	alr2209-R	CTGTCTTGGTCAGGTCTTCGGG
	
	alr2211-F	CAAGATCGCCAAGTGGTGAGGC
	
	alr2211-R	GTGTTTCCAGGTAACAAGCCCC
	
	alr2581-F	GCGGGGACAGAAGGCAAATTTG
	
	alr2581-R	CGCCTAATTGTTCTGTACCCCC
	
	alr2588-F	CAGGTGAGGCGGGATTACCTG
	
	alr2588-R	ACTCCCCCTGGTTCTAGTTGTC
	
	alr2592-F	CCCCAAAGCCAGTGGAGGGA
	
	alr2592-R	CCCCTAGCGGCTCGAACATTG
	
	alr2596-F	CGCCTGTGCGCGATATTCCTG
	
	alr2596-R	CAGGCGCAACAAATACCCGTTC
	
	alr2626-F	GGCGTTCAACCAGGGGGAGT
	
	alr2626-R	CTAAACCAGGTCTGGGTATGGC
	
	alr3242-F	CCCGACGTGATAGTGGGTCAC
	
	alr3242-R	CTGGGGGAATCCGGCTGCAT
	
	rnpB-F	AGGGAGAGAGTAGG GTTGG
	
	rnpB-R	GGTTTACCGAGCCAGTACCTCT

Sequence confirmation	all2619-20 F	CTCCCATTTCTCCGAAGCTG
	
	all2619-20 R	CAACGCTGGGGCCAACATAG
	
	alr4028-29 F	CTATGGACTTAACCAACAAAGCATTC
	
	alr4028-29 R	CTTCTCTGGTTTAAGGTCAGGATTAC

Finally, we investigated the expression pattern of TDBTs under conditions enforcing heterocyst formation by growth in medium without a nitrogen source (BG11_0_). We again analysed the amount of transcript in the four different media. Strikingly, in BG11_0 _medium 17 genes are expressed (Figure 6A, lane 6, Figure 6C, black hemicircle) but seven of them are not expressed in BG11. Moreover, we found four genes - *alr2153 *and *alr3242 *(*hutA*-like),*alr2626 *and *alr2185 *(*fhuA*-like) - for which a transcript was detected only under additional metal starvation (BG11_0 _-Fe, -Cu or -Fe/-Cu). Remarkably, *all2620 *is expressed under all conditions without a nitrogen source, which suggests that *all2620 *is not a pseudogene. In general, one can conclude that not only metal starvation but also nitrogen starvation induces transcription of TBDT-encoding genes in *Anabaena *sp. PCC 7120.

**Figure 7:**
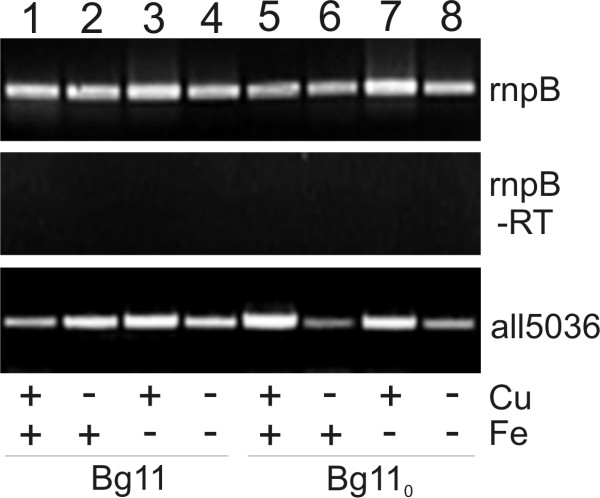
**Expression of *all5036 *encoding the only TonB in *Anabaena *sp. PCC 7120**. *Anabaena *sp. PCC 7120 were grown as for experiments shown in Figure 6 in BG11 (lane 1-4) or BG110 (lane 5-8) with and without a source of ferric ammonium citrate and/or copper and reverse transcription (RT) polymerase chain reaction was performed in the absence (-RT) or presence of reverse transcriptase on isolated RNA using primers for rnpB and all5036 (listed in Table 3) to visualize expression.

As only *all5036 *encodes a TonB-like protein in *Anabaena *sp. PCC 7120 we analysed its expression under the conditions outlined (Figure 7). As expected, *all5036 *transcript can be detected under all conditions tested. Assuming that the function of all identified TBDTs in *Anabaena *sp. depends on TonB, All5036 is required for iron homeostasis in general.

## Conclusion

By clustering ~4,600 TBDTs we found that they group by their substrate and not according to their taxonomy with the exception of regions IX, XI, XIII and C. The latter are specific for sequences from bacteroidetes and γ-proteobacteria, respectively. Hence, the transported molecule dominates the sequence variation among TBDTs. According to the occurrence of expTBDTs within clusters, we were able to assign a tentative substrate for almost two-thirds of the analysed sequences. We have developed a website for a further detailed inspection of the clustering of individual sequences . Here, the individual clusters or sequences can be highlighted based on the presentation in Figure 2. However, the current assignment has to be viewed with care as Schauer and colleagues pointed out that further substrates might be discovered in future [128], which will then be introduced into the web interface. We identified several clusters of TBDTs with putatively so far unknown substrates. Further research on a few candidate proteins of each of these clusters would be of great interest, as it would significantly advance the knowledge on substrate uptake by bacteria on the protein level and it might also reveal new potential drug targets.

Large differences to previously suggested classifications were not observed for iron-transporting TBDTs. Generally, our approach resembles previous classifications of TBDTs according to their substrates based on a smaller number of sequences and a phylogenetic tree reconstruction [53,80,82,111,112], but the positioning of the IutA and of the ViuA sequences differs with respect to distances previously proposed [82,112]. In contrast to the report by LeVier and Guerinot who placed ViuA between the lactoferrin and transferrin recognizing transporters [82], we found that ViuA (sequence 74, Region VI) clearly clusters with FyuA (sequence 72) sequences. This discrepancy might reflect the fact that: (i) more sequences of TBDTs are available nowadays; and (ii) the methodology to analyse sequence relationships has improved.

A deviation from this general picture was found for the predicted BtuBs, which are spread over a long stripe from regions II to V. Hence, BtuBs might show a similar diffuse distribution pattern like the heme and hydroxamate transporters (regions I and V, respectively). The predicted BtuBs might, therefore, transport substrates only structurally related to cobalt-complexing vitamin B_12_.

### TBDTs in *Anabaena *sp

Based on database searches, we have identified 25 sequences with TonB-box signature [39] leading to 22 sequences coding for putative TBDTs in *Anabaena *sp. PCC 7120 (Figures 3, 4, 5). Strikingly, at least five different types of transporters are identified (FhuA, ViuA, IutA, BtuB and HutA type) and this number greatly exceeds the number of genes coding for TBDTs of almost all other (sequenced) cyanobacteria with the exception of *Gloeobacter violaceus*. In turn, this is the only species for which some of the TBDTs could not be functionally assigned. As already discussed, it has been hypothesized that iron limitation might have been one of the selective forces in evolution of cyanobacteria. *Gloeobacter violaceus *(33 TBDTs) was isolated in 1972 from a calcareous rock near the Vierwaldstättersee in Switzerland, whereas *Anabaena variablis *(previously *Anabaena flos-aquae *strain A-37; 10 TBDTs) was isolated in 1964 from fresh water of the Mississippi, USA. Both strains are considered non-symbiotic. *G. violaceus *is rather unique with respect to the absence of thylakoid structure and does not form filaments [132] like *Synechocystis *sp. PCC 6803 (4 TBDTs), which was isolated from fresh water in California and deposited in the Pasteur collection in 1968 . Hence, the number of TBDTs does not correlate with filament or heterocyst formation. It might, however, correlate with the habitat from which the species were isolated, with respect to either species competition for iron or iron limitations in the environment *per se*. Therefore, symbiotic cyanobacteria such as *Nostoc punctiforme *may possibly contain a rather low number of TBDTs because iron is provided by the host. Unfortunately, to the best of our knowledge, the source of *Anabaena *sp. strain PCC 7120 - formerly named *Nostoc muscorum *ISU ([133]; further synonyms are *Anabaena *sp. ATCC 27893, *Nostoc *sp. strain PCC 7120) - is unknown and it is considered to be a 'free living cyanobacterium'. The observation that this cyanobacterium is susceptible to viruses isolated from the Lake Mendota, Dane County, Wisconsin, USA, [133] might suggest that a similar environment was its place of isolation. This would be in line with an original natural habitat of *Anabaena *sp. PCC 7120 that contained rather limited iron sources, because it has been reported that the iron concentration in rivers is higher than in lakes ([134]). The variety of TBDT classes found in *Anabaena *sp. rather agrees with iron limited environmental conditions. The only TBDT type which could not be identified in the analysed cyanobacterial species, in general, and, thereby, also in *Anabaena *sp. PCC 7120, is the FecA-type (diferric dicitrate) which can be found in many other bacteria. To date, schizokinen is the only confirmed siderophore which is secreted by *Anabaena *sp. PCC 7120 [27] and, recently, its transporter was identified [33]. However, additional siderophores are secreted by *Anabaena *sp. [33,131], but they have not yet been characterized. Nevertheless, other interpretations for the variable number of TBDTs might still be possible.

### The environment influences the expression of TBDT genes in *Anabaena *sp

In line with iron limitation in the native environment, several differential expression regulation regimes have been observed. For instance, six out of 14 genes encoding hydroxamate recognizing FhuA-like transporters are expressed under (almost) all tested conditions (Figure 6C, grey and grey dashed line, Table 1). The same holds true for one BtuB-like transporter encoded by *all3310*, which is in accordance with its identification in a proteome analysis of cells grown under standard conditions [135,136]. Interestingly, the other BtuB-like transporter encoded by the joint gene *all4028/all4029 *is only expressed under iron-limiting conditions (Figure 6C, black dotted line). Furthermore, the *iutA*-like genes are always expressed under nitrogen-limiting conditions, whereas *hutA*-like genes are only expressed upon metal starvation (Figure 6C, black dashed dotted line). Also, for the gene encoding the schizokinen transporter SchT (Alr0397) only a moderate and intermediate influence of iron starvation on expression was observed [33]. The gene encoding the only putative catecholate transporter (All4026) appears to be expressed under non-limiting conditions as well as after nitrogen starvation. To our surprise, we did not observe a transcript under iron limitation but under copper limitation in BG11 or in the absence of both metals in BG11 and BG11_0_. Such a clear relation to copper starvation was detected for four FhuA-type transporters as well (Figure 6C). The relation between the expression of genes encoding for TBDTs in *Anabaena *sp. and copper agrees with the recent observation that genes involved in siderophore production are also induced by copper starvation [131]. Nevertheless, the components of the network regulating the expression of TBDT encoding genes still need to be identified. Even though a complex network of TBDTs was discovered, only a single TonB protein was found in 58% of Gram-negative bacteria [137]. The gene is expressed under all tested conditions and, hence, it has to be considered as a master 'regulator' of the large group of TBDTs.

## Methods

### Identification of TonB-dependent transporters

Ninety-eight TBDT sequences were extracted from the NCBI database after extensive literature search. For 67 of them, experimental data is available, but for four of them the substrate is still unknown. Information on predicted substrates for the remaining 27 is available (see Additional file 1, [14-19,22,34-124]). These predictions are based on co-localization with genes of a specific metabolic pathway or on co-regulation by either transcription factors or a riboswitch [128]. Moreover, we downloaded 686 completely sequenced eubacterial genomes from the NCBI ftp server  that were available in June 2008. In order to locate putative TBDTs in the genomes, we searched for open reading frames containing the TBDT β-barrel domain and the plug domain. To this end, we used hmmsearch (hidden Markov model search) from the hmmer package  and the profile hidden Markov models PF00593 and PF07715 provided by the PFAM database [138,139]. The hmmsearch output-files were parsed considering only hits with an *E*-value < 10^-10^. We used only sequences for further analysis that resulted in a significant hit for both domains.

### Phylogenetic analysis and clustering

The 97 cyanobacterial TBDT sequences were aligned with MAFFT [140] and a maximum likelihood tree was constructed with IQPNNI v3.3.b4 [141]. As a substitution model we selected VT [142] with gamma-distributed rate heterogeneity. Support values were calculated from 1000 bootstrap replicates. The consensus tree was reconstructed with Tree-Puzzle v5.2 [143] applying the majority consensus rule. The program CLANS [144] was used to cluster the 4648 putative TBDTs detected in the complete genomes, and to visualize their degree of similarity. In CLANS we set the cut off such that only *P*-values < 10^-10 ^obtained by pairwise BLASTs were used for the CLANS-clustering. In the context of this manuscript, we use the term 'cluster' to refer to an aggregation of sequences. Each sequence in a cluster has at least one correspondent within the cluster with a BLAST p-value < 10^-90 ^leading to 195 clusters with at least two elements.

To further elucidate the relationship of the 195 clusters, we ran CLANS 100 times with a random initial configuration of the sequences in 3d space. In each run we determined the cluster centres and computed pair-wise distances between the centres. With the PHYLIP package v3.68 [145] we constructed a neighbour-joining tree for the resulting 100 distance matrices and we inferred the majority rule consensus tree with support values for the splits in the consensus tree.

### Genome loci of TonB-dependent transporters in *Anabaena *sp. PCC 7120

The annotations of genes upstream and downstream of the TBDT loci, shown in Figure 5, were done manually.

### Analysis of the operon structure

Genomic DNA of *Anabaena *sp. was isolated as described [146]. The intergenic sequences between *all2619 *and *all2620 *and between *alr4028 *and *alr4029*, respectively, and additional ~250 bp inside each flanking gene were amplified with 5' Prime PCR Extender Polymerase (5' Prime, Hamburg, Germany) according to the manufacturer's protocol. The PCR product was cloned into pCR2.1 (Invitrogen, Karlsruhe, Germany), transformed into DH5α (GibcoBRL, Eggenstein, Deutschland) and the resulting plasmids purified for sequencing.

### RNA isolation and analysis

Total RNA was isolated from 50 ml cells of log phase cultures (OD_750 _≈ 2) as described [147]. RT-PCRs were performed according to the protocol of the Invitrogen SuperScript^® ^III First-Strand Synthesis System for Random Hexamer Primers (Invitrogen, Carlsbad, USA). The used oligonucleotides are listed in Table 3.

## Abbreviations

Hmmsearch: hidden Markov model search; LbpA: lactoferrin-binding protein A; RT-PCR: reverse transcription polymerase chain reaction; TbpA: transferrin-binding protein A; TBDT: TonB-dependent transporter; expTBDT: experimentally characterized TBDT; pTBDT: TBDT with predicted substrate.

## Authors' contributions

The idea and strategy were developed by ES and AvH. OM and SS performed database searches and bioinformatic analyses. Laboratory experiments were performed by KN. ES drafted the manuscript, which was finalized by AvH and ES. All authors read and approved the final manuscript.

## Supplementary Material

Additional file 1 **List of experimentally characterized TBDTs and of TBDTs with predicted substrate used for classification.** Click here for file

Additional file 2 **Number of TBDTs detected in analyzed genomes.** Click here for file
