# Supplementary material for: TonB-dependent transporters and their occurrence in cyanobacteria
Source: BMC Biol. 2009 Oct 12;7:68. doi: 10.1186/1741-7007-7-68 (PMC2771747; doi:10.1186/1741-7007-7-68)
Supplement: Additional file 1 — List of experimentally characterized TBDTs and of TBDTs with predicted substrate used for classification. [file 1741-7007-7-68-S1.PDF]

List of experimentally characterized TBDTs and of TBDTs with predicted substrate used for classification

All TBDTs used for the analysis of the CLANs results are given. Italic indicates TBDTs with predicted substrate. The left columns give the numbers used in Figure 1, the second column the Cluster (see Figure 1A), the third column gives the assigned name, the fourth column the GenBank ID, the fifth column the source species, the sixth column the identified siderophores recognized by the according protein, the seventh column the siderophore classification and the eighth column representative references for the substrate and substrate classification (o.a.: only annotated). '[metal]' in column 6 indicates that the type of transported metal ion is known, but the according metallophore has not yet been identified.

No.	Cluster	Name	GenBank	Species	Substrate	Siderophore/Substrate Classification	Ref.
1	11	OprC	1498191	Pseudomonas aeruginosa	Copper chelate	Unknown	[34]
2	12	BfeA	538279	Bordetella pertussis	enterobactin	Catecholate	[35, 36]
3	12	PirA	2981053	Pseudomonas aeruginosa	enterobactin	Catecholate	[36, 37]
4	12	PfeA	548479	Pseudomonas aeruginosa	enterobactin	Catecholate	[36, 38]
5	12	FepA	2507463	Escherichia coli K12	enterobactin	Catecholate	[36, 39]
6	12	IroN	2738252	Salmonella enterica	salmochelin	glycosylated Catecholate	[40, 41]
7	12	CfrA	112360090	Campylobacter jejuni	enterobactin	Catecholate	[36, 42]
8	12	CirA	2507462	Escherichia coli K12	2,3-dihydroxybenzoylserine(DHBS)	Catecholate-Carboxylate	[43, 44]
9	12	IrgA	12644182	Vibrio cholerae	Enterobactin	Catecholate	[36, 45]
10	12	BfrA	1314835	Bordetella bronchiseptica	2,3-dihydroxybenzoylserine(DHBS)	Catecholate-Carboxylate	[44, 46]
11	18	HutR	147671724	Vibrio cholerae	haem	Porphyrine	[47]
12	18	HuvA	12697532	Listonella anguillarum	haem	Porphyrine	[48]
13	18	HutA	529727	Vibrio cholerae	haem	Porphyrine	[49]
14	18	PhuR	3044098	Pseudomonas aeruginosa	haem	Porphyrine	[37]
15	18	PfhR	4838477	Pseudomonas fluorescens	haem	Porphyrine	[37]
16	132	HpuB	11386826	Neisseria meningitidis	haem	Porphyrine	[50]
17	108	HmbR	687640	Neisseria meningitidis	haem	Porphyrine	[51]
18	17	HgbA	28194090	Actinobacillus pleuropneumoniae	haem	Porphyrine	[52]
19	86	HemR	6016198	Yersinia enterocolitica	haem	Porphyrine	[53]
20	86	HmuR	2501236	Yersinia pestis	haem	Porphyrine	[54]
21	86	ChuA	1763009	Escherichia coli O157:H7	haem	Porphyrine	[55]
22	86	ShuA	1655877	Shigella dysenteriae	haem	Porphyrine	[56]
23	86	HxuC	1170441	Haemophilus influenzae	haem	Porphyrine	[57]
24	48	TdhA	33151615	Haemophilus ducreyi 35000HP	haem	Porphyrine	[58]
25	152	HasR	34787214	Serratia marcescens	haem	Porphyrine	[59]

No.	Cluster	Name	GenBank	Species	Substrate	Siderophore/Substrate Classification	Ref.
26	107	MhuA	50403825	Moraxella catarrhalis	haem	Porphyrine	[60]
27	15	FetA_FrpB	4768684	Neisseria gonorrhoeae	enterobactin	Catecholate	[36,61]
28	59	VctA	18476494	Vibrio cholerae	enterobactin	Catecholate	[36, 62]
29	16	LbpA	915278	Neisseria gonorrhoeae	lactoferrin	Fe(III)-binding protein	[22, 63]
30	16	TbpA	150361	Neisseria gonorrhoeae	transferrin	Fe(III)-binding protein	[22, 64]
31	112	FrpB4	15646121	Helicobacter pylori 26695	[Nickel]	unknown	[14]
32	4	BtuB	416728	Escherichia coli K12	Vitamin B12	Porphyrine	[65]
33	*4*	*XCC3067*	*21232497*	*Xanthomonas campestris pv. campestris str. ATCC 33913*	*Vitamin B12*	Porphyrine	[66]
34	*4*	*PA1271*	*15596468*	*Pseudomonas aeruginosa PAO1*	*Vitamin B12*	Porphyrine	[66]
35	*4*	*BPSL0976*	*53718618*	*Burkholderia pseudomallei K96243*	*Vitamin B12*	Porphyrine	[66]
36	*4*	*RS02718*	*17547119*	*Ralstonia solanacearum GMI1000*	*Vitamin B12*	Porphyrine	[66]
37	*165*	*CC1750*	*109897435*	*Pseudoalteromonas atlantica T6c*	*Vitamin B12*	Porphyrine	[66]
38	*4*	*VC0156*	*15640186*	*Vibrio cholera*	*Vitamin B12*	Porphyrine	[66]
39	*160*	*RSP_2402*	*77462960*	*Rhodobacter sphaeroides 2.4.1*	*Vitamin B12*	Porphyrine	[66]
40	40	MxcH	162452159	Sorangium cellulosum 'So ce 56'	Myxochelin	Catecholate	[67, 68]
41	10	IutA	1170593	Escherichia coli	aerobactin	Citrate-Hydroxamate	[69, 70]
42	10	RhtA	6685883	Sinorhizobium meliloti	Rhizobactin 1021	Citrate-Hydroxamate	[71, 72]
43	*166*	*SO_0815*	*24372404*	*Shewanella oneidensis MR-1*	*Vitamin B12*	Porphyrine	[66]
44	82	RumA	1247762	Morganella morganii	ferric rhizoferrin	Carboxylate	[73, 74]
45	82	FecA	729471	Escherichia coli K12	diferric dicitrate	Citrate	[75, 76]
46	*25*	*PA2911*	*15598107*	*Pseudomonas aeruginosa PAO1*	*Vitamin B12*	Porphyrine	[66]
47	*25*	*RPA0407*	*39933484*	*Rhodopseudomonas palustris CGA009*	*Vitamin B12*	Porphyrine	[66]
48	0	VciA	147673813	Vibrio cholerae O395	unknown	unknown	[77]
49	0	PiuA_Fiu	115587765	Pseudomonas aeruginosa	pyochelin	Phenolate	[78, 79]
50	0	FoxA	1169726	Yersinia enterocolitica	desferrioxamine	Hydroxamate	[80, 81]
51	0	FegA	1518696	Bradyrhizobium japonicum	desferrioxamine	Hydroxamate	[82, 81]
52	0	FctA	871032	Erwinia chrysanthemi	chrysobactin	Catecholate	[83, 84]
53	0	FmtA	53719389	Burkholderia pseudomallei K96243	ferric malleobactin	Hydroxamate	[85, 86]
54	0	OrbA	76810798	Burkholderia pseudomallei	ferric ornibactin	Citrate-Hydroxamate	[87, 88][a]
55	0	FhuA	2507464	Escherichia coli K12	ferrichrome	Hydroxamate	[89, 90]
56	0	OptS	116050410	Pseudomonas aeruginosa	desferrioxamine	Hydroxamate	[81, 91]
57	0	BfrI	33592999	Bordetella pertussis Tohama I	unknown	unknown	o.a.
58	-	BfrZ	6850914	Bordetella bronchiseptica	unknown	unknown	[92]
59	-	PrhA	17549099	Ralstonia solanacea rum	transducer without transport function	unknown	[93]
60	6	PbuA	1172035	Pseudomonas sp. M114	pseudobactin M114	Citrate-Catecholate-Hydroxamate	[94, 95]
61	6	FpvA	12230910	Pseudomonas aeruginosa	pyoverdine	Catecholate-Hydroxamate	[96, 97]
62	6	PupA	45723	Pseudomonas putida WCS358	pseudobactin A	Citrate-Catecholate-Hydroxamate	[95, 98]
63	6	PupB	585759	Pseudomonas putida WCS358	pseudobactin A	Citrate-Catecholate-Hydroxamate	[95, 99]
64	6	FauA	4589285	Bordetella pertussis	alcaligin	Hydroxamate	[100, 101]
65	6	FhuE	2507465	Escherichia coli K12	Coprogen, ferrioxamine B, rhodoturolic acid	Hydroxamates	[102-105]
66	6	FptA	1169730	Pseudomonas aeruginosa	pyochelin	Phenolate	[79, 106]
67	*9*	*Bcep18194_b2436*	*78063283*	*Burkholderia sp. 383*	*Thiamin*	*Vitamin B1*	[107]

No.	Cluster	Name	GenBank	Species	Substrate	Siderophore/Substrate Classification	Ref.
68	*9*	*XCC0674*	*21230149*	*X. campestris pv. Campestris str.* ATCC 33913	*Thiamin*	*Vitamin B1*	[107]
69	7	FatA	132510	Listonella anguillarum	anguibactin	Catecholate-Hydroxamate	[108, 109]
70	7	BauA	49175779	Acinetobacter baumannii	anguibactin	Catecholate-Hydroxamate	[109, 110]
71	7	FcuA	1169655|	Yersinia enterocolitica	anguibactin	Catecholate-Hydroxamate	[111, 109]
72	79	FyuA	517234	Yersinia enterocolitica	yersiniabactin	Phenolate	[112, 113]
73	79	IrpC	17380443	Yersinia pestis	yersiniabactin	Phenolate	[113, 114]
74	140	ViuA	267356	Vibrio cholerae	vibriobactin	Catecholate	[115, 116]
75	*118*	*SO_2715*	*24374256*	*Shewanella oneidensis MR-1*	*Thiamin*	*Vitamin B1*	[107]
76	*118*	*CPS_0067*	*71281279*	*Colwellia psychrerythraea 34H*	*Thiamin*	*Vitamin B1*	[107]
77	45	SftP	6019468	Pseudomonas putida	hexylsulfate		[117]
78	64	SuxA	21232787	Xanthomonas campestris pv. campestris str. ATCC 33913	sucrose	disaccharide	[15]
79	*64*	*Sfri_3988*	*114565138*	*Shewanella frigidimarina* NCIMB 400	*sucrose*	*disaccharide*	[15]
80	*52*	*bll6948*	*27382059*	*Bradyrhizobium japonicum* USDA 110	*[Nickel]*	*Unknown*	
81	*52*	*Daro_1684*	*71907314*	*Dechloromonas aromatica*RCB	*[Cobalt]*	*Unknown*	[118]
82	*52*	*Daro_3944*	*71909555*	*Dechloromonas aromatic* RCB	*[Nickel]*	*Unknown*	[118]
83	*9*	*BF0615*	*53711906*	*Bacteroides fragilis* YCH46	*Thiamin*	*Vitamin B1*	[107]
84	*9*	*PG1899*	*34541505*	*Porphyromonas gingivalis* W83	*Thiamin*	*Vitamin B1*	[107]
85	26	RagA	110636973	Cytophaga hutchinsonii ATCC 33406	digested proteins	Polypeptides	[19][b]
86	26	RagA	110636966	Cytophaga hutchinsonii ATCC 33406	digested proteins	Polypeptides	[19][b]
87	26	SusC	29349110	Bacteroides thetaiotaomicron VPI-5482	Malto-oligo-saccharides /starch	Oligo-/Polysaccharides	[18]
88	26	CsuF	29348741	Bacteroides thetaiotaomicron VPI-5482	Chondroitin sulfate / hyaluronic acid	unbranched polysaccharides (GlcA GalNAc) / unbranched polymer of N-acetyl-glucosamine + glucuronic acid	[17, 119, 120]
89	26	OmpW	29348978	Bacteroides thetaiotaomicron VPI-5482	unknown	unknown	[121]
90	63	MalA	16126526	Caulobacter crescentus CB15	Maltodextrins	starch hydrolysate	[16]
91	*63*	*SO_3514*	*24375018*	*Shewanella oneidensis MR-1*	*Chito-oligosaccharides*	N-acetylglucosamine oligomer	[122, 123]
92	*63*	*Sden_2708*	*91794059*	*Shewanella denitrificans* OS217	*Chito-oligosaccharides*	N-acetylglucosamine oligomer	[122, 123]
93	*63*	*CPS_1021*	*71281574*	*Colwellia psychrerythraea* 34H	*Chito-oligosaccharides*	N-acetylglucosamine oligomer	[122, 123]
94	*63*	*CC_0446*	*16124701*	*Caulobacter crescentus* CB15	*Chito-oligosaccharides*	N-acetylglucosamine oligomer	[122, 123]
95	*63*	*XCC0120*	*21229598*	*X. campestris pv. campestris str.* ATCC 33913	*Pectin*	heteropolysaccharide	[15]
96	*63*	*XCC2944*	*21232375*	*X. campestris pv. campestris str.* ATCC 33913	*Chito-oligosaccharides*	N-acetylglucosamine oligomer	[122, 123]
97	*72*	*XCC4120*	*21233542*	*X. campestris pv. campestris str.* ATCC 33913	*Xylan*	heteropolysaccharide	[15]
98	41	BF1991	53713281	Bacteroides fragilis YCH46	Fibronectin	extracellular matrix glycoprotein	[124]

[a]…we have marked one sequence (54; annotated as OrbA in GenBank) with 79% similarity and 67% identity to the one described in the reference (*Burkholderia cepacia*, gi|11230853, [87]); sequences with a higher similarity/identity are in the same cluster, too.

[b]…we have marked two sequences (85, 86; annotated as RagA in GenBank) with 65% similarity and 31% identity to the one described in the reference (*Porphyromonas gingivalis* W83, gi|34540042, [19]).
