# Supplementary material for: TonB-dependent transporters and their occurrence in cyanobacteria
Source: BMC Biol. 2009 Oct 12;7:68. doi: 10.1186/1741-7007-7-68 (PMC2771747; doi:10.1186/1741-7007-7-68)
Supplement: Additional file 2 — Number of TBDTs detected in analyzed genomes. [file 1741-7007-7-68-S2.PDF]

Number of TBDTs detected in analyzed genomes

Species names are given in columns 1 and 3, the number of identified TBDTs in the genome of the according species in columns 2 and 4, respectively. All species without TBDTs are not listed.

Species	TBDTs
Acaryochloris marina MBIC11017	12
Acidiphilium cryptum JF-5	3
Acidobacteria bacterium Ellin345	4
Acidovorax avenae subsp. citrulli AAC00-1	14
Acidovorax sp. JS42	8
Acinetobacter baumannii	28
Acinetobacter baumannii ATCC 17978	10
Acinetobacter sp. ADP1	25
Actinobacillus pleuropneumoniae	1
Actinobacillus pleuropneumoniae L20	4
Actinobacillus pleuropneumoniae serovar 3 str. JL03	5
Actinobacillus succinogenes 130Z	1
Aeromonas hydrophila subsp. hydrophila ATCC 7966	10
Aeromonas salmonicida subsp. salmonicida A449	10
Agrobacterium tumefaciens str. C58	8
Alcanivorax borkumensis SK2	10
Alkalilimnicola ehrlichei MLHE-1	8
Anabaena sp. PCC 7120	22
Anabaena variabilis ATCC 29413	10
Anaeromyxobacter dehalogenans 2CP-C	2
Anaeromyxobacter sp. Fw109-5	3
Aquifex aeolicus VF5	1
Arcobacter butzleri RM4018	17
Azoarcus sp. BH72	20
Azoarcus sp. EbN1	6
Azorhizobium caulinodans ORS 571	13
Bacteroides fragilis NCTC 9343	73
Bacteroides fragilis YCH46	76
Bacteroides thetaiotaomicron VPI-5482	108
Bacteroides vulgatus ATCC 8482	74
Bartonella bacilliformis KC583	1
Bartonella henselae str. Houston-1	1
Bartonella quintana str. Toulouse	1
Bartonella tribocorum CIP 105476	1
Bdellovibrio bacteriovorus HD100	3
Beijerinckia indica subsp. indica ATCC 9039	12
Bordetella avium 197N	10
Bordetella bronchiseptica	2
Bordetella bronchiseptica RB50	18
Bordetella parapertussis 12822	13

Species	TBDTs
Bordetella pertussis	2
Bordetella pertussis Tohama I	15
Bordetella petrii DSM 12804	12
Bradyrhizobium japonicum	1
Bradyrhizobium japonicum USDA 110	10
Bradyrhizobium sp. BTAi1	13
Bradyrhizobium sp. ORS278	8
Brucella abortus biovar 1 str. 9-941	3
Brucella abortus S19	3
Brucella canis ATCC 23365	2
Brucella melitensis 16M	3
Brucella melitensis biovar Abortus 2308	3
Brucella ovis ATCC 25840	3
Brucella suis 1330	3
Brucella suis ATCC 23445	3
Burkholderia ambifaria AMMD	15
Burkholderia ambifaria MC40-6	14
Burkholderia cenocepacia AU 1054	20
Burkholderia cenocepacia HI2424	23
Burkholderia cenocepacia MC0-3	23
Burkholderia mallei ATCC 23344	8
Burkholderia mallei NCTC 10229	8
Burkholderia mallei NCTC 10247	8
Burkholderia mallei SAVP1	7
Burkholderia multivorans ATCC 17616	14
Burkholderia phymatum STM815	3
Burkholderia pseudomallei 1106a	10
Burkholderia pseudomallei 1710b	10
Burkholderia pseudomallei 668	10
Burkholderia pseudomallei K96243	10
Burkholderia sp. 383	27
Burkholderia thailandensis E264	10
Burkholderia vietnamiensis G4	10
Burkholderia xenovorans LB400	13
Campylobacter concisus 13826	2
Campylobacter curvus 525.92	9
Campylobacter fetus subsp. fetus 82-40	4
Campylobacter jejuni RM1221	2
Campylobacter jejuni subsp. doylei 269.97	4
Campylobacter jejuni subsp. jejuni 81116	2
Campylobacter jejuni subsp. jejuni 81-176	2

Species	TBDTs
Campylobacter jejuni subsp. jejuni NCTC 11168	3
Candidatus Blochmannia floridanus	1
Candidatus Blochmannia pennsylvanicus str. BPEN	1
Caulobacter crescentus CB15	59
Caulobacter sp. K31	83
Chlorobium chlorochromatii CaD3	1
Chlorobium phaeobacteroides DSM 266	3
Chlorobium tepidum TLS	5
Chromobacterium violaceum ATCC 12472	12
Chromohalobacter salexigens DSM 3043	9
Citrobacter koseri ATCC BAA-895	22
Colwellia psychrerythraea 34H	35
Cupriavidus taiwanensis	2
Cyanothece sp. ATCC 51142	1
Cytophaga hutchinsonii ATCC 33406	9
Dechloromonas aromatica RCB	12
Delftia acidovorans SPH-1	37
Desulfococcus oleovorans Hxd3	1
Desulfotalea psychrophila LSv54	1
Desulfovibrio desulfuricans G20	1
Desulfovibrio vulgaris subsp. vulgaris DP4	1
Desulfovibrio vulgaris subsp. vulgaris str. Hildenborough	1
Dinoroseobacter shibae DFL 12	3
Enterobacter sakazakii ATCC BAA-894	8
Enterobacter sp. 638	13
Erwinia carotovora subsp. atroseptica SCRI1043	18
Erwinia chrysanthemi	1
Erythrobacter litoralis HTCC2594	19
Escherichia coli	1
Escherichia coli 536	16
Escherichia coli APEC O1	15
Escherichia coli ATCC 8739	9
Escherichia coli CFT073	19
Escherichia coli E24377A	9
Escherichia coli HS	8
Escherichia coli K12	6
Escherichia coli O157:H7	1
Escherichia coli O157:H7 EDL933	14
Escherichia coli O157:H7 str. Sakai	13
Escherichia coli SECEC SMS-3-5	13
Escherichia coli str. K-12 substr. DH10B	10
Escherichia coli str. K-12 substr. MG1655	9
Escherichia coli UTI89	18
Escherichia coli W3110	9
Flavobacterium johnsoniae UW101	57
Flavobacterium psychrophilum JIP02/86	8
Fusobacterium nucleatum subsp. nucleatum ATCC 25586	5
Geobacter metallireducens GS-15	2
Geobacter sulfurreducens PCA	1
Geobacter uraniireducens Rf4	2

Species	TBDTs
Gloeobacter violaceus PCC 7421	32
Gluconacetobacter diazotrophicus PAl 5	20
Gluconobacter oxydans 621H	13
Gramella forsetii KT0803	28
Granulibacter bethesdensis CGDNIH1	5
Haemophilus ducreyi 35000HP	2
Haemophilus influenzae	1
Haemophilus influenzae 86-028NP	5
Haemophilus influenzae PittEE	4
Haemophilus influenzae PittGG	4
Haemophilus influenzae Rd KW20	8
Haemophilus somnus 129PT	5
Haemophilus somnus 2336	5
Hahella chejuensis KCTC 2396	8
Halorhodospira halophila SL1	8
Helicobacter acinonychis str. Sheeba	6
Helicobacter hepaticus ATCC 51449	4
Helicobacter pylori 26695	5
Helicobacter pylori HPAG1	6
Helicobacter pylori J99	6
Herminiimonas arsenicoxydans	9
Hyphomonas neptunium ATCC 15444	39
Idiomarina loihiensis L2TR	29
Jannaschia sp. CCS1	1
Janthinobacterium sp. Marseille	31
Klebsiella pneumoniae subsp. pneumoniae MGH 78578	13
Leptospira biflexa serovar Patoc strain 'Patoc 1 (Paris)'	5
Leptospira borgpetersenii serovar Hardjo-bovis JB197	6
Leptospira borgpetersenii serovar Hardjo-bovis L550	6
Leptospira interrogans serovar Copenhageni str. Fiocruz L1-130	9
Leptospira interrogans serovar Lai str. 56601	11
Leptothrix cholodnii SP-6	7
Listonella anguillarum	2
Magnetococcus sp. MC-1	2
Magnetospirillum magneticum AMB-1	2
Mannheimia succiniciproducens MBEL55E	3
Maricaulis maris MCS10	21
Marinobacter aquaeolei VT8	4
Marinomonas sp. MWYL1	15
Mesorhizobium loti MAFF303099	1
Mesorhizobium sp. BNC1	3
Methylibium petroleiphilum PM1	15
Methylobacillus flagellatus KT	21
Methylobacterium extorquens PA1	15
Methylobacterium radiotolerans JCM 2831	20
Methylobacterium sp. 4-46	8
Methylococcus capsulatus str. Bath	5
Moraxella catarrhalis	1
Morganella morganii	1

Species	TBDTs
Myxococcus xanthus DK 1622	11
Neisseria gonorrhoeae	3
Neisseria gonorrhoeae FA 1090	6
Neisseria meningitidis	2
Neisseria meningitidis 053442	6
Neisseria meningitidis FAM18	10
Neisseria meningitidis MC58	9
Neisseria meningitidis Z2491	8
Nitratiruptor sp. SB155-2	2
Nitrobacter hamburgensis X14	11
Nitrobacter winogradskyi Nb-255	13
Nitrosococcus oceani ATCC 19707	8
Nitrosomonas europaea ATCC 19718	29
Nitrosomonas eutropha C91	12
Nitrosospira multiformis ATCC 25196	8
Nodularia spumigena CCY9414	2
Nostoc punctiforme PCC 73102	2
Novosphingobium aromaticivorans DSM 12444	66
Ochrobactrum anthropi ATCC 49188	8
Opitutus terrae PB90-1	8
Parabacteroides distasonis ATCC 8503	66
Paracoccus denitrificans PD1222	22
Parvibaculum lavamentivorans DS-1	7
Pasteurella multocida subsp. multocida str. Pm70	12
Pelobacter carbinolicus DSM 2380	6
Pelobacter propionicus DSM 2379	14
Pelodictyon luteolum DSM 273	3
Photobacterium profundum SS9	7
Photorhabdus luminescens subsp. laumondii TTO1	12
Polaromonas naphthalenivorans CJ2	3
Polaromonas sp. JS666	5
Polynucleobacter sp. QLW-P1DMWA-1	3
Porphyromonas gingivalis W83	7
Prosthecochloris vibrioformis DSM 265	2
Pseudoalteromonas atlantica T6c	62
Pseudoalteromonas haloplanktis TAC125	35
Pseudomonas aeruginosa	6
Pseudomonas aeruginosa PA7	30
Pseudomonas aeruginosa PAO1	35
Pseudomonas aeruginosa UCBPP-PA14	37
Pseudomonas entomophila L48	30
Pseudomonas fluorescens	1
Pseudomonas fluorescens Pf-5	43
Pseudomonas fluorescens PfO-1	26
Pseudomonas mendocina ymp	17
Pseudomonas putida	2
Pseudomonas putida F1	30
Pseudomonas putida GB-1	47
Pseudomonas putida KT2440	30
Pseudomonas putida W619	22

Species	TBDTs
Pseudomonas putida WCS358	1
Pseudomonas sp. M114	1
Pseudomonas stutzeri A1501	13
Pseudomonas syringae pv. phaseolicola 1448A	21
Pseudomonas syringae pv. syringae B728a	19
Pseudomonas syringae pv. tomato str. DC3000	25
Psychrobacter arcticus 273-4	1
Psychrobacter cryohalolentis K5	4
Psychrobacter sp. PRwf-1	8
Psychromonas ingrahamii 37	1
Ralstonia eutropha H16	17
Ralstonia eutropha JMP134	10
Ralstonia metallidurans CH34	16
Ralstonia solanacearum GMI1000	15
Rhizobium etli CFN 42	2
Rhizobium leguminosarum bv. viciae 3841	3
Rhodobacter sphaeroides 2.4.1	4
Rhodobacter sphaeroides ATCC 17025	3
Rhodobacter sphaeroides ATCC 17029	7
Rhodoferax ferrireducens T118	3
Rhodopseudomonas palustris BisA53	8
Rhodopseudomonas palustris BisB18	10
Rhodopseudomonas palustris BisB5	7
Rhodopseudomonas palustris CGA009	18
Rhodopseudomonas palustris HaA2	17
Rhodospirillum rubrum ATCC 11170	13
Roseobacter denitrificans OCh 114	1
Saccharophagus degradans 2-40	43
Salinibacter ruber DSM 13855	17
Salmonella enterica	1
Salmonella enterica subsp. arizonae serovar 62:z4 z23:--	8
Salmonella enterica subsp. enterica serovar Choleraesuis str. SC-B67	8
Salmonella enterica subsp. enterica serovar Paratyphi A str. ATCC 9150	6
Salmonella enterica subsp. enterica serovar Paratyphi B str. SPB7	8
Salmonella enterica subsp. enterica serovar Typhi str. CT18	6
Salmonella enterica subsp. enterica serovar Typhi Ty2	6
Salmonella typhimurium LT2	8
Serratia marcescens	1
Serratia proteamaculans 568	16
Shewanella amazonensis SB2B	23
Shewanella baltica OS155	28
Shewanella baltica OS185	34
Shewanella baltica OS195	38
Shewanella denitrificans OS217	21
Shewanella frigidimarina NCIMB 400	26
Shewanella halifaxensis HAW-EB4	18
Shewanella loihica PV-4	20
Shewanella oneidensis MR-1	23

Species	TBDTs
Shewanella pealeana ATCC 700345	24
Shewanella putrefaciens CN-32	29
Shewanella sediminis HAW-EB3	21
Shewanella sp. ANA-3	34
Shewanella sp. MR-4	35
Shewanella sp. MR-7	35
Shewanella sp. W3-18-1	27
Shewanella woodyi ATCC 51908	39
Shigella boydii CDC 3083-94	8
Shigella boydii Sb227	8
Shigella dysenteriae	1
Shigella dysenteriae Sd197	8
Shigella flexneri 2a str. 2457T	4
Shigella flexneri 2a str. 301	4
Shigella flexneri 5 str. 8401	6
Shigella sonnei Ss046	10
Silicibacter sp. TM1040	1
Sinorhizobium medicae WSM419	4
Sinorhizobium meliloti	1
Sinorhizobium meliloti 1021	8
Solibacter usitatus Ellin6076	5
Sorangium cellulosum 'So ce 56'	10
Sphingomonas wittichii RW1	140
Sphingopyxis alaskensis RB2256	32
Sulfurimonas denitrificans DSM 1251	5
Sulfurovum sp. NBC37-1	2
Synechococcus sp. JA-2-3B'a(2-13)	2
Synechococcus sp. JA-3-3Ab	2
Synechococcus sp. PCC 7002	6
Synechocystis sp. PCC 6803	4
Syntrophobacter fumaroxidans MPOB	3
Syntrophus aciditrophicus SB	2
Thiobacillus denitrificans ATCC 25259	7
Thiomicrospira crunogena XCL-2	5
Verminephrobacter eiseniae EF01-2	6

Species	TBDTs
Vibrio cholerae	4
Vibrio cholerae O1 biovar eltor str. N16961	6
Vibrio cholerae O395	9
Vibrio fischeri ES114	8
Vibrio harveyi ATCC BAA-1116	10
Vibrio parahaemolyticus RIMD 2210633	11
Vibrio vulnificus CMCP6	7
Vibrio vulnificus YJ016	7
Wolinella succinogenes DSM 1740	11
Xanthobacter autotrophicus Py2	3
Xanthomonas axonopodis pv. citri str. 306	66
Xanthomonas campestris pv. campestris str. 8004	65
Xanthomonas campestris pv. campestris str. ATCC 33913	64
Xanthomonas campestris pv. vesicatoria str. 85-10	52
Xanthomonas oryzae pv. oryzae KACC10331	31
Xanthomonas oryzae pv. oryzae MAFF 311018	33
Xylella fastidiosa 9a5c	9
Xylella fastidiosa M12	9
Xylella fastidiosa M23	9
Xylella fastidiosa Temecula1	9
Yersinia enterocolitica	4
Yersinia enterocolitica subsp. enterocolitica 8081	11
Yersinia pestis	2
Yersinia pestis Angola	11
Yersinia pestis Antiqua	11
Yersinia pestis biovar Microtus str. 91001	11
Yersinia pestis CO92	11
Yersinia pestis KIM	12
Yersinia pestis Nepal516	11
Yersinia pestis Pestoides F	11
Yersinia pseudotuberculosis IP 31758	11
Yersinia pseudotuberculosis IP 32953	12
Yersinia pseudotuberculosis YPIII	11
Zymomonas mobilis subsp. mobilis ZM4	17
